# Comparison of transepithelial resistance measurement techniques: Chopsticks vs. Endohm

**DOI:** 10.1186/s12575-017-0053-6

**Published:** 2017-05-10

**Authors:** Rebecca A. Sheller, Maria E. Cuevas, Maria C. Todd

**Affiliations:** Biology Department, Southwestern University, 1001 E University Ave, Georgetown, TX 78626 USA

**Keywords:** Claudins, Tight junctions, MDCK, MCF-7, MDA-MB-157, PC inserts, PET inserts

## Abstract

**Background:**

TER measurements across confluent cellular monolayers provide a useful indication of TJ strength between epithelial and endothelial cells in culture. Having a reliable and accurate method of measuring cell-to-cell adhesion is critical to studies in pathophysiology and cancer metastasis. However, the use of different technical approaches to measure TER has reportedly yielded inconsistent measurements within the same cell lines.

**Methods:**

In the current study, we compared the peak TER values for the MDCK (canine kidney) and MCF-7 (human breast cancer) epithelial cell lines using two common approaches (Chopstick and Endohm) and two types of polymer inserts (PC and PET).

**Results:**

Both cell lines demonstrated a statistically significant difference in the peak TERs obtained using the two different approaches. Further, the MDCK (but not the MCF-7) cells demonstrated a statistically significant difference between the peak TERs when using the same approach but different inserts.

**Conclusion:**

Our study indicates the importance of using a single approach when seeking to measure and compare the TER values of cultured cell lines.

## Background

Transepithelial resistance (TER) values of cultured epithelial cell monolayers provide an indication of tight junction (TJ) strength [1, 2]. TJs are responsible for the majority of paracellular resistance in the interstitial space between epithelial cells [1, 3]. There are at least 40 different proteins found in TJs including the transmembrane proteins claudins, occludin, tricellulin and junctional adhesion molecules. The claudin family consists of 24 proteins that are expressed in a cell type dependent manner. Through homotypic and heterotypic cell-cell interactions, claudins specify both the ion permeability and TER between cells [1]. In addition, changes in the ectopic expression of claudin proteins have been reported to affect the TER of both normal and tumor cell lines [4].

When epithelial cells are seeded onto culture well inserts (permeable supports) at a relatively high density, the monolayer develops polarity and TJs form within several days [5]. When using chopstick electrodes to measure TERs, investigators place one electrode in an upper, apical compartment and another electrode in a lower, basal compartment. A voltohmmeter passes current between the two electrodes and measures the voltage difference between the electrodes to calculate the resistance across the monolayer [5]. Chopstick electrodes allow researchers to make TER measurements without removing the inserts upon which the cellular monolayer grows. When using an Endohm chamber to measure TERs, each insert must be removed from its culture well and placed into the Endohm recording chamber. Endohm measurements have been reported to provide more reliable TER data as the device creates a more uniform current density, providing an average resistance for a large area of the epithelial monolayer [6]. In contrast, chopstick electrodes only measure the current across a small sample of the monolayer. Notably, Zhang et al. [7] reported more reproducible TER values of porcine brain microvessel endothelial cells using an Endohm chamber rather than the handheld chopstick electrodes.

Both chopstick and Endohm techniques measure TERs for monolayers on either polycarbonate (PC) or polyester (PET) inserts. The major drawback of using PC inserts is that the investigator cannot visualize the cells in the culture wells and thus cannot correlate TER values with the degree of cellular confluency. Use of the PET inserts, however, allows visualization of the growing cells via phase contrast microscopy.

The goal of the current study was to compare the TER values obtained from the analysis of mammalian epithelial cell lines using the chopstick electrodes versus Endohm chamber. In addition, we evaluated effects of insert polymer type (PC versus PET) on the TER values. We used the following cell lines in our study: MDCK (a cell line previously reported to develop strong TJs with consistently high TERs [4, 8, 9]; MCF-7 (metastatic adenocarcinoma breast cancer cells that form tightly adherent epithelial monolayers but express abnormally elevated levels of the TJ proteins, claudin-3 and -4) [10] and MDA-MB-157 (metastatic adenocarcinoma breast cancer cells that form only loosely adherent monolayers with a mesenchymal-like appearance).

We found a statistically significant difference between the values obtained using the chopstick electrodes compared to the Endohm chamber in the MDCK and MCF-7 cell lines that formed TJs. The MDA-MB-157 cell line, not surprisingly based on their morphology, did not form TJs. In the case of the MCF-7 cells, we did not find a statistically significant difference in TER values when PC or PET inserts were used in conjunction with either technique. However, the difference in TER values obtained from the analysis of MDCK cells grown on PC or PET inserts was statistically significant using both techniques.

## Results

**Fig. 1:**
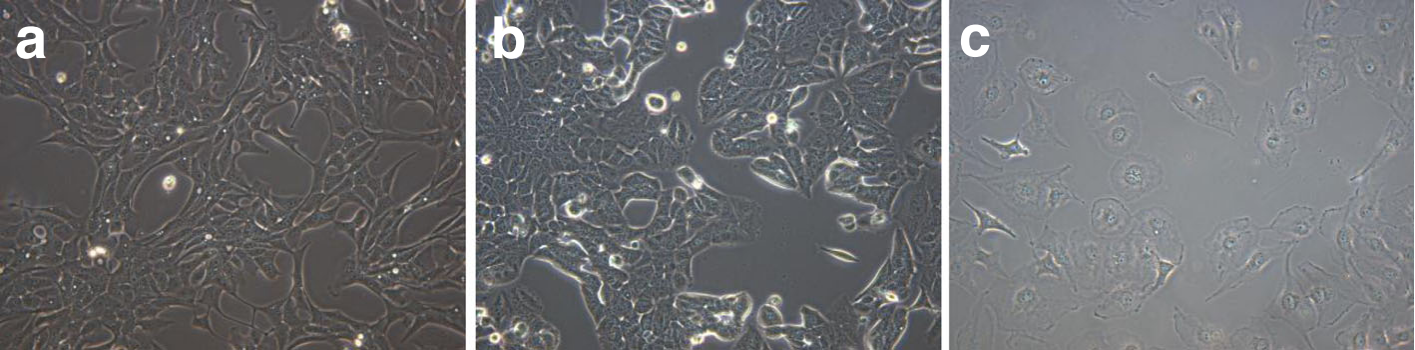
Phase-contrast micrographs of mammalian epithelial cell lines: MDCK (**a**), MCF-7 (**b**) and MDA-MB-157 (**c**) cells were cultured in their respective media and photographed with a phase-contrast microscope at 10× (MDCK) and 20× (MCF-7 and MDA-MB-157) magnification

The objective of the current study was to compare the chopstick versus Endohm chamber techniques for measuring the TER of cultured mammalian cells. To this end we used the following cell lines: MDCK kidney epithelial cells, MCF-7 and MDA-MB-157 breast cancer cell lines (Fig. 1).

The cells were seeded at a density of 2–4 × 10^5^ cells per insert into 12-well plates. Using both the Chopsticks and Endohm methods, we observed negligible TER values, barely above background, at 24 h post-seeding of the MCF-7 cells (post-seeding day 1). On days 2 and 3, slight elevations in TERs were observed. Within 4–6 days after seeding, MCF-7 cultures developed peak TERs that averaged 402 Ω x cm^2^ (Chopsticks) and 435 Ω x cm^2^ (Endohm) on PC filters. Similarly, in the same time frame using PET filters MCF-7 developed peak TERs that averaged 424 Ω x cm^2^ (Chopsticks) and 462 Ω x cm^2^ (Endohm). These data represent 4 independent experiments and 16 individual TER measurements (Table 1). When we used a paired t-test to compare peak TER values obtained using the Chopstick versus Endohm method there was a statistical difference for cells plated on PC (*p* ≤ 0.01) and PET (*p* ≤ 0.02) inserts. In contrast, no statistical difference was observed when we compared peak TER values obtained with PC versus PET inserts using the same approach (*p* ≥ 0.4 for both Chopstick and Endohm).

**Table 1:** Comparison of TER measurements using the Chopstick vs Endohm techniques

Cell Line	Chopstick TER ($\overset{-}{x}$ ± SEM Ω x cm^2^)		Endohm TER ($\overset{-}{x}$ ± SEM Ω x cm^2^)	
Substrate	PC	PET	PC	PET
MCF-7 (*n* = 16)	402 ± 28^a*^	424 ± 12^b*^	435 ± 31^a*^	462 ± 16^b*^
MDCK (*n* = 5)	7049 ± 355^c**^	5280 ± 352^d**^	8055 ± 263^c**^	6431 ± 361^d**^
MDA-MB-157 (*n* = 7)	7 ± 1	13 ± 4	1 ± 0.5	6 ± 0.2

Values represent means ± standard error of the mean. Levels of significance were tested separately for each of the cell lines using a paired *t*-test. ^a^ *p* ≤ 0.01 when comparing MCF-7’s TER values using PC insert but different techniques. ^b^ *p* ≤ 0.02 when comparing MCF-7’s TER values using PET insert but different techniques. * No significant difference between peak TER values obtained when MCF-7 ells were seeded on PC versus PET inserts using the same approach (*p* ≥ 0.4 for both Chopstick and Endohm). ^c^ *p* = 0.04 when comparing MDCK’s TER values using PC insert but different techniques; ^d^ *p* = 0.0003 MDCK’s TER values level of significance when comparing different techniques using PET insert. **Statistically significant differences when comparing MDCK’s peak TER values obtained with PC versus PET inserts using the same approach (Chopstick *p* = 0.009 and Endohm *p* = 0.002). No statistical analysis was done for MDA-MB-157 cells as they did not form functional TJs

Notably, regardless of the approach used to measure TER, the MDCK cells showed about 13-fold higher peak TER using PET inserts and approximately 18-fold higher peak TER using PC inserts than the MCF-7 cells, indicative of the exceptionally strong TJs formed by the MDCK cells (Table 1). When we used a paired-t test to compare peak TER values using Chopstick versus Endohm method we observed a significant difference when using PC (*p* = 0.04) and PET inserts (*p* = 0.0003). In contrast with the MCF-7 cells we observed statistically significant differences when comparing peak TER values obtained with PC versus PET inserts using the same approach (Chopstick *p* = 0.009 and Endohm *p* = 0.002).

In contrast, to the above MCF-7 and MDCK data, the MDA-MB-157 cells did not form functional TJs over the course of the experiment.

## Discussion

Measurement of TER is frequently used to determine the strength of TJs between epithelial and endothelial cells in culture. However, the use of different technical approaches to measure TER sometimes results in inconsistent reports for TER readings within the same cell lines. For example, when using chopstick electrodes the TER values obtained were higher in A6 renal cells [11], MDCK strain 1 and LLCK1 porcine kidney cells [12] than those obtained using Endohm chambers. These higher values may be attributable to incorrect positioning of the chopstick electrodes, resulting in the subjection of cells to a non-uniform electrical field [11].

To address this disparity, the goal of the current study was to compare two frequently used approaches (Chopstick and Endohm) and two types of polymer inserts (PC and PET) to measure the TER values of three mammalian cell lines (MDCK, MCF-7 and MDA-MB-157). MDCK and MCF-7 cell lines have previously been reported to form strong TJ connections [4, 13]. In contrast, the MDA-MB-157 cells form very loose connections in culture and therefore acted as a negative control for TJ formation.

We obtained TERs for MCF-7 cells of over 400 Ω x cm^2^ when grown on PC or PET inserts using both the chopsticks and Endohm approaches. However, there was a statistically significant difference between the peak TERs obtained using the two different approaches. The peak TERs that we observed for MCF-7 cells are consistent with reports by Somasiri et al. [13] who obtained TER values of 497 Ω x cm^2^, but are in contrast to reports by Martin et al. [14] and Li et al. [15] who reported markedly lower values of 20–40 Ω x cm^2^ for MCF-7 cells. Notably, both Somasiri et al. [13] and Li et al. [15] used the same millicell electrical resistance system but obtained values that differed by 10-fold. These inconsistencies demonstrate the need for an approach to measuring TER that is reliably accurate and consistent.

In keeping with the reportedly high TER values associated with MDCK cells we were not surprised to observe dramatically lower peak TER values in MCF-7 cells relative to MDCK cells [8, 9]. As we observed in the MCF-7 cells, there was a statistically significant difference between the peak TER values obtained in MDCK cells using the Chopsticks vs Endohm approaches. However, unlike the MCF-7 cells, when using the same TER measurement method but different inserts, we observed a statistically significant difference between the peak TER values in the MDCK cells.

Peak TER values vary among different cell lines due to cell type, size and shape, junctional protein expression, and differential expression of TJ proteins [1, 16–18]. Notably, both the MDCK and MCF-7 cells in our study display epithelial cell morphology whereas the MDA-MB-157 cells have a transitional morphology between that of epithelial and mesenchymal cells. The latter is responsible for the lack of cell-to-cell connectivity demonstrated by the MDA-MB-157 cells in culture. In addition, the MCF-7 cells overexpress claudin-3 [10] relative to the normal levels expressed by MDCK and MDA-MB-157 cells. The abnormally high levels of claudin-3 expressed by the MCF-7 cells may contribute to the weakening of the TJs in these cells (as reflected by their lower TER values relative to MDCK cells).

In addition to the measurement apparatus and type of insert (PC or PET) TER measurements for any specific cell line can vary due to confounding factors such as calcium concentration, temperature fluctuation, cellular confluence prior to seeding and seeding density onto inserts [1, 5, 11, 19–22]. We have attempted to address these factors by culturing the three cell lines in media containing the same calcium concentration at a constant temperature. In addition, we grew all three cells lines to a high degree of confluency prior to seeding onto PC or PET inserts. Seeding was performed within a narrow range 2–4 × 10^5^ cells/insert.

## Conclusion

We have demonstrated that the TER values obtained for the MDCK cells (that form strong TJs) are both apparatus and insert dependent. In contrast, the TER values obtained for the MCF-7 cells that form weaker TJ than those of MDCK cells are only apparatus dependent. Our study indicates the importance of using a single approach when seeking to measure and compare the TER values of cultured cell lines. In addition, when comparing TER data in the primary literature it is critical to be aware of the method and inserts use to obtained TER values for a particular cell line.

## Methods

### Culture conditions

MCF-7 and MDA-MB-157 breast cancer cells and Madin-Darby Canine Kidney cells (MDCK; NBL-2; parental cell line) were purchased from the American Type Culture Collection (ATCC, Manasas, VA). MCF-7 and MDA-MB-157 cells were grown in Minimum Essential Medium (MEM) and MDCK cells were grown in Eagle’s Minimum Essential Medium (EMEM). Both media were supplemented with 10% FBS and 1% penicillin/streptomycin/2 mM glutamine (PSG). Cultures were maintained in a humidified incubator at 37 °C and 5% CO_2_. Cells were passaged using 0.25% trypsin/1 mM EDTA.

### TER measurements

Polycarbonate (PC) or polyester (PET) transwell inserts (0.4 μm, 1.2 cm) (Corning, Tewksbury, MA) were placed in 12-well plates and incubated with MEM or EMEM supplemented with 10% FBS and PSG for one hour at 37 °C. Cells harvested from flasks at a confluency of 80–95% were seeded at a density of 2–4 × 10^5^ cells/inserts. Medium was changed daily for the duration of each experiment.

TER measurements were taken daily, beginning 24 h post-seeding, in a laminar flow hood at room temperature using the manual chopstick electrodes (World Precision Instruments, Sarasota, FL) or an Endohm chamber in conjunction with an EVOM epithelial voltometer (World Precision Instruments). The chopstick electrodes and the Endohm chamber were sterilized by rinsing them with 70% ethanol followed by sterile PBS. When using the handheld chopstick silver/silver chloride electrode, the shorter end of the electrode was placed in the upper chamber while the longer end was placed in the bottom chamber of the well and the electrode was held in position until a TER reading was obtained. In contrast, when using the Endohm chamber, the insert was removed with forceps and placed in the Endohm chamber containing a lower, concentric silver/silver chloride electrode. An electrode cap was then placed on top of the cells and the TER measurement was taken without having to hold a measuring device. For both methods TER measurements were recorded in duplicate. To obtain TERs in Ω × cm^2^ the background resistance (taken from an insert with media only) was subtracted from the average TER reading per well, and the subsequent value multiplied by the surface area of the filter (1.12 cm^2^).

### Statistics

Data were reported as the mean ± SEM. Paired-t-test was used to compare Chopstick versus Endohm methods using the same filter (PC or PE) and to compare TER measurements between PC and PE using the same method. A *p* value of ≤0.05 was considered statistically significant.

## Abbreviations

PC: Polycarbonate
PET: Polyester
TER: Transepithelial resistance
TJ: Tight junction
